# Commentary: Person-centred care to overcome barriers to self-care among patients with chronic obstructive pulmonary disease

**DOI:** 10.1177/17449871261442801

**Published:** 2026-06-07

**Authors:** Ann Ekdahl, Sara Lundell

**Affiliations:** Department of Community Medicine and Rehabilitation, Umeå University, Umeå, Sweden; Department of Community Medicine and Rehabilitation, Umeå University, Umeå, Sweden

**Commentary to:** Barriers to self-care in chronic obstructive pulmonary disease in China: a qualitative study (Liu et al., 2026).

Chronic obstructive pulmonary disease (COPD) continues to represent a major global health burden, affecting patients, relatives and healthcare systems. Self-care in COPD is a highly important part of non-pharmacological treatment, which alleviates symptoms, prevents deterioration, hospitalisations and high healthcare costs (Fabbri et al., 2023; Global Initiative for Chronic Obstructive Lung Disease, 2025).

The study by Liu et al., 2026 aimed to explore barriers to self‑care maintenance, monitoring and management among patients with COPD in China. The findings fit within the theoretical foundations of person‑centred care (PCC), a connection we will focus on that is suitable to guide chronic illness care (Ekman et al., 2011; Olsson et al., 2013). PCC is defined as recognising the patient as a person with their own narrative, preferences and resources. It includes building an equal partnership in which the patient is actively engaged in decisions about their care that is suitable to guide chronic illness care (Ekman et al., 2011; Olsson et al., 2013). Furthermore, we will comment on the study findings (Liu et al., 2026) in relation to e-health as a PCC support and to previous research.

The purpose of the study by Liu et al. (2026) was to generate evidence that can inform targeted clinical nursing interventions and offer insights relevant to public health policy and chronic disease management. A descriptive qualitative design was employed, and semi‑structured interviews were conducted with 16 patients with COPD at a rehabilitation centre. Participant characteristics showed a sample with mostly men, and mainly mild-to-moderate symptoms. Using a deductive qualitative content analysis guided by the Middle‑Range Theory of Self‑care of Chronic Illness (Riegel et al., 2012), the analysis resulted in 7 categories and 21 subcategories.

*Obstacles to self-care maintenance* were presented as difficulties in sustaining healthy lifestyle practices and to engage in physical activity. Barriers such as limited motivation, weather conditions or family responsibility contributed to other priorities and overshadowed own rehabilitation needs. Liu et al. (2026) identified several challenges of self-care, such as variability in patients’ health literacy, healthcare professionals limited understanding of individuals’ everyday life circumstances, insufficient follow‑up in primary care and an imbalance between medical interpretations and the patients’ subjective illness experiences. The authors concluded that these challenges could increase the risk for worsened symptoms, exacerbation and unnecessary use of healthcare.

*Obstacles to self-care monitoring* were presented as underlying issues, including limited awareness of the value of symptom monitoring, reduced somatic sensitivity, lack of monitoring equipment, challenges in operating digital tools and uncertainty about interpreting health data. Such factors became a barrier of acting in a timely manner upon signs of deterioration. In addition to the lack of access to equipment, inconvenience, age barriers and resistance to learning how to operate digital tools were noted as barriers.

*Obstacles to self-care management* were presented as challenges underpinned by a range of deeper issues, including insufficient knowledge, low self-efficacy, concerns about the competence or motives of healthcare professionals and structural limitations such as cultural factors within healthcare services. Such challenges caused uncertainty and fear of making incorrect choices, resulting in deterioration. Moreover, negative experiences were described when consulting healthcare professionals, such as feelings of discomfort in hospital environments, fearing infections and the humid environment.

A key contribution of the study by Liu et al. (2026) is how it illuminates barriers to self‑care that is directly relevant for the fully adaption of PCC. Identified obstacles such as variability in patients’ health literacy highlight a gap where PCC is core. By exposing these disconnects, the study underscores the importance of strengthening PCC practices to better support patients’ self‑care capacity. For instance, PCC could be a support in addressing the barrier of sustaining a healthy lifestyle. Hansen et al. (2024) demonstrated that PCC interventions for people with COPD, particularly those incorporating tailored support and motivation for physical activity, can enhance functional capacity. Moreover, PCC has shown to strengthen patients’ self‑efficacy, support goal‑setting and foster a trusting patient–professional relationship which can enhance medication adherence among patients with COPD (Zimmermann et al., 2026).

Based on the study findings, we can also see a potential for e-health solutions, that can effectively support PCC at a distance (Ali et al., 2020). The possibility of using e-health is particularly relevant in the context of demographic shifts, increasing multimorbidity and workforce shortages, all which place pressure on healthcare organisations globally (Ibrahim et al., 2022; Salmi et al., 2025). In the study by Liu et al. (2026), participants expressed challenges in using digital tools. This can however differ between cultures. In Sweden, a study showed that 85% of people with COPD used the Internet daily. They reported that it was highly likely that they would use COPD‑adapted digital tools (Sönnerfors et al., 2021). However, people stress that e‑health tools should be seen as a complement to existing support, leading to a combination of face‑to‑face and digital interventions (Sönnerfors et al., 2023). Successful implementation of e-health tools requires training and time for learning for both professionals and patients (Marklund et al., 2021; Nyberg et al., 2023), as well as careful attention to barriers that may limit accessibility or sustained use. This reflects ongoing concerns in the broader literature regarding digital exclusion and variable health literacy among patients with long-term illnesses (Salmi et al., 2025). Tools such as mobile applications, web portals and electronic surveys, integrated with electronic health records, have the potential to streamline patient information management and facilitate collaborative, well‑informed decision‑making across long-term illnesses (Ibrahim et al., 2022).

From a theoretical and methodological viewpoint, the study is well-reasoned and uses deductive analysis in a structured and transparent way to apply the Middle‑Range Theory of Self‑care of Chronic Illness theory. The theoretical anchoring enhances the study’s explanatory depth and supports its possible translation into clinical practice. However, the barriers of self-care could be further elaborated, for example regarding stigma-related emotions in connection to smoking (Kalucza et al., 2025; Lundell et al., 2020; Woo et al., 2021). Although the sample is small and situated within a specific cultural context, the insights are nevertheless partly transferable, offering valuable knowledge for understanding how barriers can be transformed into facilitators for enhanced self‑care for patients with COPD. Besides the result in this study, the lack of cohesive interdisciplinary collaboration, limited managerial knowledge of learning processes and ethical considerations related to care decision‑making are also noted as significant barriers (Martin and Sibbald, 2022).

Overall, this study is sound and careful. In an envisioned future, the multiprofessional team supporting patients with COPD can benefit from age-friendly e-health tools and relatives to provide motivational and somatic awareness support. In such an envisioned future, healthcare professionals work with fully adopted PCC clinical practice. Future research could explore self‑care barriers in a larger and more heterogenous sample, especially given the mild symptoms and male predominance in this sample, to clarify how PCC strategies can be better tailored. In the next step, it would also be interesting to see exploration of self‑management as a broader, person‑centred construct to better understand how patients with COPD navigate and integrate this in their everyday lives.

## References

[bibr1-17449871261442801] Ali L Wallström S Barenfeld E , et al. (2020) Person-centred care by a combined digital platform and structured telephone support for people with chronic obstructive pulmonary disease and/or chronic heart failure: study protocol for the PROTECT randomised controlled trial. BMJ Open 10: e036356. 10.1136/bmjopen-2019-036356 PMC7371144 32690519

[bibr2-17449871261442801] Ekman I Swedberg K Taft C , et al. (2011) Person-centered care—ready for prime time. European Journal of Cardiovascular Nursing 10: 248–251. 21764386 10.1016/j.ejcnurse.2011.06.008

[bibr3-17449871261442801] Fabbri LM Celli BR Agustí A , et al. (2023) COPD and multimorbidity: recognising and addressing a syndemic occurrence. The Lancet Respiratory Medicine 11: 1020–1034. 37696283 10.1016/S2213-2600(23)00261-8

[bibr4-17449871261442801] Global Initiative for Chronic Obstructive Lung Disease. (2025) Global strategy for the diagnosis, management and prevention of chronic obstructive pulmonary disease: 2025 GOLD report [Online]. Available at: https://goldcopd.org/2025-gold-report/ (accessed 20 february 2026)

[bibr5-17449871261442801] Hansen TS Poulsen I Nørholm V , et al. (2024) Nutritional support and physical activity intervention programs with a person-centred approach in people with chronic obstructive pulmonary disease: A scoping review. International Journal of Chronic Obstructive Pulmonary Disease 19: 2193–2216. 39371918 10.2147/COPD.S458289 PMC11456268

[bibr6-17449871261442801] Ibrahim MS Mohamed Yusoff H Abu Bakar YI , et al. (2022) Digital health for quality healthcare: A systematic mapping of review studies. Digital Health, 8: 20552076221085810. 10.1177/20552076221085810 PMC8943311 35340904

[bibr7-17449871261442801] Kalucza S Coe AB Hajdarevic S , et al. (2025) Associations between self-conscious emotions and sociodemographic factors, lifestyle, burden of disease, and healthcare interventions: A cross-sectional study with people with chronic obstructive pulmonary disease. Respiratory Medicine 250: 108539. 41308800 10.1016/j.rmed.2025.108539

[bibr8-17449871261442801] Liu et al. (2026) Barriers to self-care in chronic obstructive pulmonary disease in China: a qualitative study. Journal of Research in Nursing. Epub ahead of print 14 May, 2026. DOI: 10.1177/17449871261428434. PMC13179310 42146842

[bibr9-17449871261442801] Lundell S Wadell K Wiklund M , et al. (2020) Enhancing confidence and coping with stigma in an ambiguous interaction with primary care: A qualitative study of people with COPD. COPD 17: 533–542. 32981381 10.1080/15412555.2020.1824217

[bibr10-17449871261442801] Marklund S Tistad M Lundell S , et al. (2021) Experiences and factors affecting usage of an eHealth tool for self-management among people with chronic obstructive pulmonary disease: Qualitative study. Journal of Medical Internet Research 23: e25672. 10.2196/25672 PMC8122287 33929327

[bibr11-17449871261442801] Martin J Sibbald S. (2022) Using patient engagement to inform the delivery of equitable care for patients with COPD and other chronic diseases – a literature review. International Journal of Integrated Care (IJIC) 22: 1–2.

[bibr12-17449871261442801] Nyberg A Sondell A Lundell S , et al. (2023) Experiences of using an electronic health tool among health care professionals involved in chronic obstructive pulmonary disease management: Qualitative analysis. JMIR Human Factors 10: e43269. 10.2196/43269 PMC10131608 36995743

[bibr13-17449871261442801] Olsson LE Jakobsson Ung E Swedberg K , et al. (2013) Efficacy of person-centred care as an intervention in controlled trials–a systematic review. Journal of Clinical Nursing 22: 456–465. 23231540 10.1111/jocn.12039

[bibr14-17449871261442801] Riegel B Jaarsma T Strömberg A (2012) A middle-range theory of self-care of chronic illness. Advances in Nursing Science 35: 194–204. 22739426 10.1097/ANS.0b013e318261b1ba

[bibr15-17449871261442801] Salmi EM Basile FW Khan FA , et al. (2025) Facilitators and barriers affecting the implementation of e-health for chronic respiratory diseases in remote settings: a qualitative evidence synthesis. BMC Health Services Research 25: 19. 39754241 10.1186/s12913-024-12050-4 PMC11699737

[bibr16-17449871261442801] Sönnerfors P Skavberg Roaldsen K Lundell S , et al. (2023) Preferences for an eHealth tool to support physical activity and exercise training in COPD: a qualitative study from the viewpoint of prospective users. BMC Pulmonary Medicine 23: 65. 36782155 10.1186/s12890-023-02353-3 PMC9925217

[bibr17-17449871261442801] Sönnerfors P Skavberg Roaldsen K Ståhle A , et al. (2021) Access to, use, knowledge, and preferences for information technology and technical equipment among people with chronic obstructive pulmonary disease (COPD) in Sweden. A cross-sectional survey study. BMC Pulmonary Medicine 21: 185. 34112150 10.1186/s12911-021-01544-4 PMC8191435

[bibr18-17449871261442801] Woo S Zhou W Larson JL . (2021) Stigma experiences in people with chronic obstructive pulmonary disease: An integrative review. International journal of chronic obstructive pulmonary disease 16: 1647–1659. 34113096 10.2147/COPD.S306874 PMC8187000

[bibr19-17449871261442801] Zimmermann M Kroppen D Ammous O , et al. (2026) COPD-patients’ perspective on adherence to therapy and its integration into a systematic literature review. BMC Pulmonary Medicine 26: 27. 41526889 10.1186/s12890-026-04102-8 PMC12849637

